# Enhancement of dissipated energy by large bending of an organic single crystal undergoing twinning deformation†

**DOI:** 10.1039/c8ra02499e

**Published:** 2018-06-14

**Authors:** Sajjad Husain Mir, Yuichi Takasaki, Emile R. Engel, Satoshi Takamizawa

**Affiliations:** Department of Materials System Science, Graduate School of Nanobioscience, Yokohama City University 22-2 Seto, Kanazawa-ku Yokohama Kanagawa 236-0027 Japan staka@yokohama-cu.ac.jp; Kanagawa Institute of Industrial Science and Technology Shimoimaizumi 705-1 Ebina Kanagawa 243-0435 Japan

## Abstract

We demonstrate exceptional twinning deformation in a molecular crystal upon application of mechanical stress. Crystal integrity is preserved and the deformation is associated with a large bending angle (65.44°). This is a new strategy to increase the magnitude of the dissipated energy in an organic solid comparable to that seen in alloys. By X-ray crystallographic analysis it was determined that a large molecular rearrangement at the twinning interface preserves the crystal integrity. Drastic molecular rearrangement at the twinning interface helps to preserve hydrogen bonding in the molecular rotation, which facilitates the large bending angle. The maximum shear strain of 218.81% and dissipated energy density of 1 MJ m^−3^ can significantly enhance mechanical damping of vibrations.

## Abstract 2

We demonstrate exceptional twinning deformation in a molecular crystal upon application of mechanical stress.

## Graphical abstract

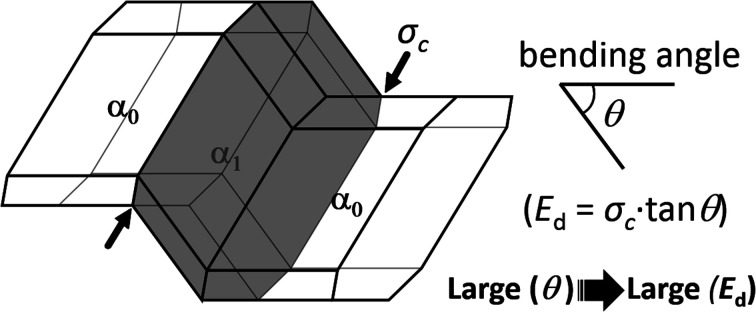

## Introduction

Twinning deformation is a type of plastic deformation that has been extensively investigated in metal alloys, mostly from the perspective of physics and materials science.^1^ There are only a few examples of twinning deformation reported for organic crystals. This could be due to the typical fragility and tiny size of organic crystals. The dissipated energy (*E*_d_) derived from twinning deformation of alloys and organic single crystals depends on the following factors: (i) coercive stress (*σ*_c_) and (ii) bending angle (*θ*), given as (*E*_d_ = *σ*_c_ tan *θ*). In alloys the required magnitude of *σ*_c_ is larger than in the case of molecular crystals, deriving a significant magnitude of *E*_d_. However, the value of *θ* is usually smaller in alloys than in organic crystals. The *E*_d_ value in alloys (usually 10^3^ to 10^4^ kJ m^−3^) is much higher than in typical organic solids (approximately 10^2^ kJ m^−3^). Application of a relatively smaller *σ*_c_ in the deformation of organic single crystals can produce a large *θ* without crystal cleavage. Achievement of a large *θ* paves the way for new design principles to increase the value of *E*_d_ in organic single crystals. Moreover, alloys have polycrystalline properties so on average *θ* is reduced due to their isotropic nature, whereas, in organic single crystals the composition is almost anisotropic and associated with comparatively higher values of *θ* which is advantageous for achieving enhancing *E*_d_.

Some of the reports of twinning deformation in single crystals relate to organometallic compounds such as ferrocene^2^ and tetramethyle-tetraselenafulvalene (TMTSF)_2_X^3^ (X = ClO_4_, PF_6_, AsF_6_, and NO_3_), while among organic solids the following examples have been reported: 1,3,5-tribromo-2,4,6-triiodobenzene,^4^ 1,3,5-trichloro-2,4,6-triiodobenzene,^4^l-lysine monohydrochloride dihydrate,^5^ and adipic acid doped with 3-methyl adipic acid.^6^ Studies of twinning deformation in such organic or organometallic solids are underdeveloped in contrast to the studies in metallic solids^7^ involving a control of their mechanical durability or deformability.

Recently, our research group has reported some stress-induced twinning deformation in organic single crystals,^8–11^ supported with experimental evidence by microscopic observations, X-ray crystal structure analysis, and force measurements. For example, the organosuperelastic crystals of the planar molecule 3,5-difluorobenzoic acid^8^ showed exceptional twinning. Crystal of 5-chloro-2-nitroaniline^9^ exhibited twinning ferroelasticity. The flexible rod shaped adipic acid molecule showed ferroelasticity driven by conformational change.^10^ Finally, a non-planar 4,4′-dicarboxydiphenyl ether^11^ exhibited ferroelasticity by partial ring flipping. However, the bending angles in these crystals were 27.8°, 49.21°, 44.65°, and 16.9°, respectively. Moreover, the *E*_d_ value of these crystals is not so high as compared to that of alloys. Herein, we investigated twinning deformation in a single crystal of 2-methyl-5-nitro-benzoic acid (C_7_H_8_NO_4_). Our results indicate exceptional mechanical twinning with an unprecedentedly large bending angle and large coercive stress, which requires drastic molecular rearrangement at the twinning interface to preserve the hydrogen bonding and avoid crystal cleavage. These factors contribute towards a large *E*_d_, which is an important property for mechanical damping of vibrations.

## Results and discussion

**Scheme 1:**
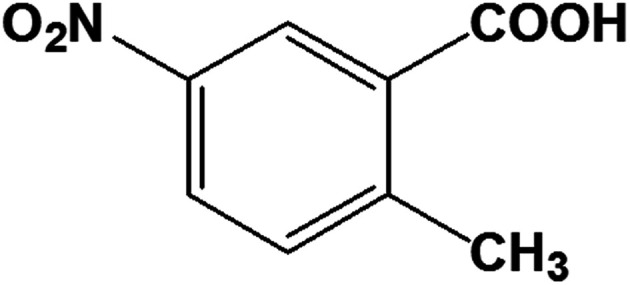
Molecular structure of 2-methyl-5-nitrobenzoic acid (1).

2-Methyl-5-nitrobenzoic acid (C_7_H_8_NO_4_) (1) (Scheme 1) is a beige colored aromatic compound with a high melting point of 184 °C. 1 is an important simple compound serving as a raw material for pharmaceuticals, agrochemicals, and dyes. Well-shaped, pale yellow colored rods with typical lengths of 0.2–1.0 mm and thicknesses of 0.1–0.3 mm were obtained by recrystallization of 1 from acetone at 298 K.

**Fig. 1:**
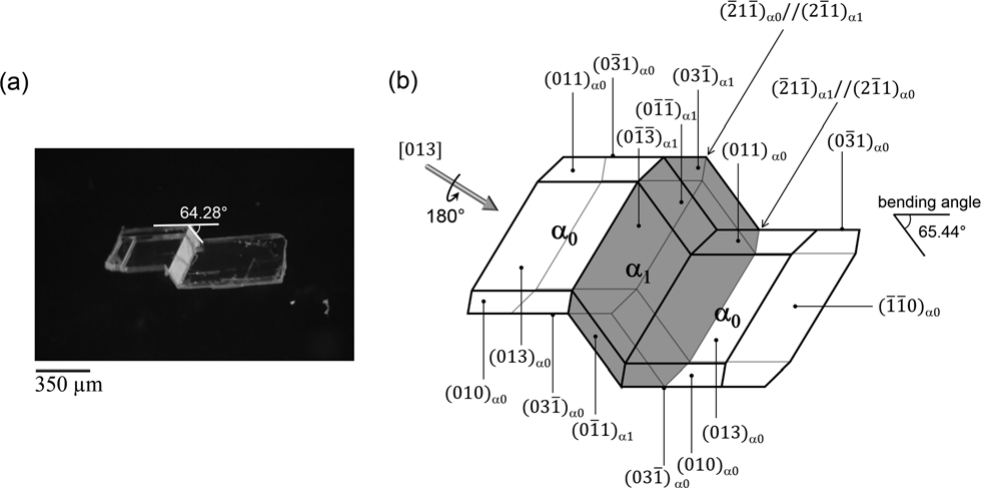
(a) Optical image of the twinned crystal, prepared by compression on crystal surface (110)_α_0__, [013] and (b) crystal face indices of the mechanically twinned crystal.

Twinning deformation was induced in a single crystal by the application of compression stress perpendicular to (110)_α_0__ and (1̄1̄0)_α_0__ (mother domain: α_0_) crystal faces at 298 K. The crystal deformed in a diffusionless manner (Fig. 1a). The bending angle is estimated to be 65.44° between α_0_ and α_1_ (α_1_: twinned domain), which corresponds to the angle between (03̄1)_α_0__ and (031̄)_α_1__, which agrees well with the value 64.28° obtained by measurement from the optical microscope (Fig. 1a). It is surprising that such a large bending can be achieved without considerably damaging the crystal.

**Fig. 2:**
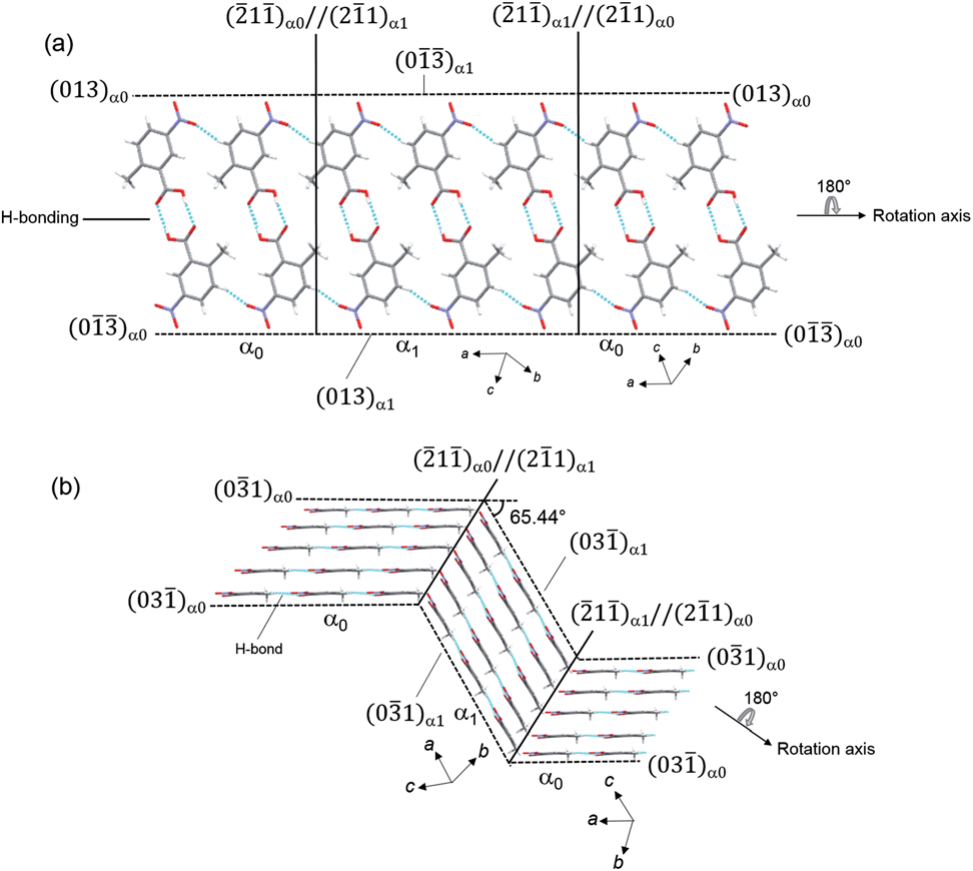
Crystal packing diagram projected along (a) (013)_α_0__ and (b) (03̄1)_α_0__.

Single-crystal X-ray structure analysis confirmed the twinning deformation under shear force. Both the α_0_ and α_1_ crystal structures have an identical crystal structure with the same symmetry *i.e.* triclinic crystal system and *P*1̄ space group^12^ (Table S1†). According to the indices of crystal faces in the bent shape as shown in Fig. 1b, the twinning interface is (2̄11̄)_α_0__//(21̄1)_α_1__. The α_1_ domain is related to α_0_ domain by a 180° rotation about the axis along [013]_α_0__ (Fig. 1b). The deformation requires drastic molecular rearrangement when the interface passes through the molecule. Considering the lattice correspondence require to achieve the bent crystal shape, the continuity of hydrogen bonding across the interface connecting α_0_ and α_1_ domains should be ensured by adjustment of the molecular orientations facing at the interface and the strength of hydrogen bonding between molecules at the interface. Molecules at the twining interface are surrounded by four other neighboring molecules, that keeps hydrogen bonding stronger on passing of rotational plane. We presume this facilitates large bending without crystal cleavage. A similar speculation was indicated in the elastic curvature of an organic single crystal.^13^ Typical O–H⋯O hydrogen bonding between carboxylic acid groups occurs along [013] with the C–O distance of 2.639 Å (intermolecular dotted lines in Fig. 2b). In addition, there are C–H⋯O interactions along [03̄1] between the aromatic hydrogen H and O on the nitro group with C–O distance of 2.673 Å. The combination of O–H⋯O and C–H⋯O hydrogen bonds constitutes 2D arrangement in the plane of (013)_α_0__. The hydrogen bonding between carboxylic acid groups is the dominant force over weak van der Waals interactions between nitro and aromatic hydrogen atoms.

The maximum strain of 1 is expected to be 218.81% from tan*θ*, where *θ* is the bending angle. This calculated maximum strain is higher than for previous reports on organic crystals that have comparatively smaller bending angles than 1. For example, the strain values for (TMTSF)_2_ClO_4_,^14*a*^ (TMTSF)_2_PF_6_,^14*c*^ 3,5 difluorobenzoic acid,^8^ and 5-chloro-2-nitroaniline^9^ are 33.3%, 36%, 52.7% and 115.9%, respectively. Moreover, the strain value is higher than for certain twinned metals. For examples, the values for gold nanopillars^15*a*^ and copper nanowires^15*b*^ are 5% and 7.2%, respectively.

**Fig. 3:**
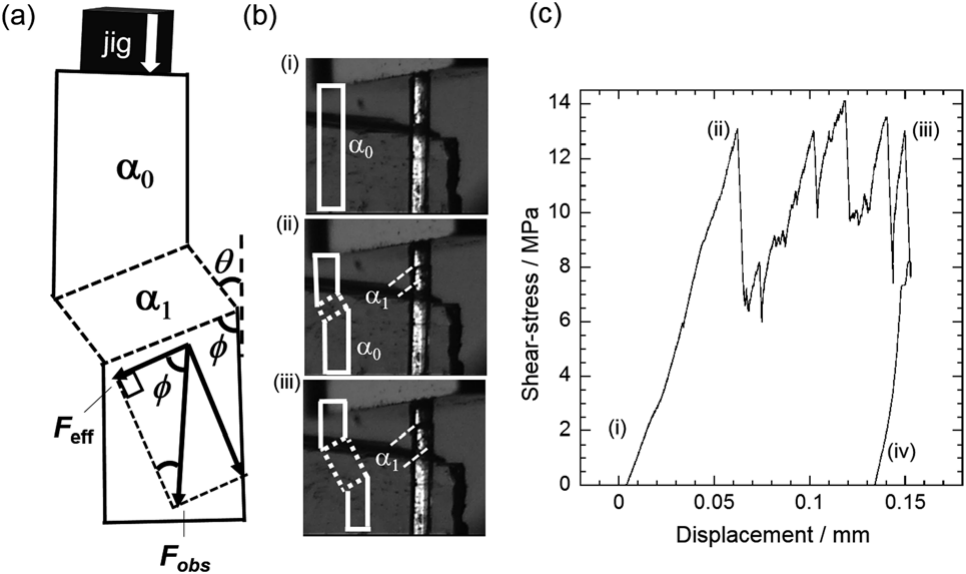
Measurement of stress–strain curve, (a) cartoon illustration of crystal deformation pattern and force components, (b) snapshots of the twinning deformation of shear-stress (i–iii) (Movie S1†) with inset sketches of the deformation pattern, and (c) stress–strain curve at 298 K.

We also conducted compression measurements of a single-crystal specimen. Despite the large bending angle and the brittleness of the crystal we could successfully induce twinning by loading stress along the long axis of the crystal (Fig. 3a). We prevented crystal breakage by avoiding stress perpendicular to the crystal surface. Because we presume if stress is perpendicular to the surface, components of the stress are in opposite direction at the interface, thats causes seperation of the crystal at the twining interface. Whereas, compression causes effective stress components in the direction as shown in Fig. 3a that prevents crystal breakage at the interface on twinning deformation. Therefore, a stress–strain curve was obtained by compressing the crystal *via* the crystal surface (110)_α_0__ (Fig. 3a), which derives the effective force along the angle 57.28° between twinning interface and effective shear. The effective stress that causes twining deformation was derived by applying the formula (*F*_eff_/cross-sectional area of the α_0_/α_1_ boundary), *i.e.* (13.40 × 10^−15^ m^2^) (Fig. 3a). One end of a single-crystal was fixed to a glass stage with a glue. A glass jig was then pushed against the (110)_α_0__ crystal surface at a constant speed of 30 μm min^−1^ (ESI, Movie S1†). As shown in Fig. 3c, stress was detected after the glass jig reached the crystal surface and an increase in the loading force began (3c(i and ii)). The effective stress reached 6.891 MPa at which point the twinning interface was generated (3c(ii)). The coercive stress achieved is the largest amongst all reports.^8–11^ The α_1_ domain started to grow from both sides of the crystal. The growth of the α_1_ domain was not smooth, producing spikes (3c(ii and iii)) in the curve. This could be ascribed to the generation of the multiple domains. On holding the displacement of the jig, the α_1_ remained present and strain was recorded by removing the stress (iii and iv). The estimated dissipated strain energy was calculated as 1000 kJ m^−3^ (0.6796 kJ kg^−1^, and 123.109 J mol^−1^). Based on the equation (*E*_d_ = *σ*_c_ tan *θ*), the *E*_d_ value of 1 is 65.24 times larger than the corresponding value for 3,5-difluorobenzoic acid *i.e.* 15.47 kJ m^−3^ (0.010 kJ kg^−1^, 1.642 J mol^−1^),^8^ and 4.67 times larger than in the case of 5-chloro-2-nitroaniline *i e.* 216 kJ m^−3^ (0.136 kJ kg^−1^, 23.46 J mol^−1^).^9^ This large value of *E*_d_ is comparable to that of alloys and much higher than typical for organic solids. Since *E*_d_ is a derivative of the applied shear stress (*σ*) and deformation angle (*θ*) of the crystal, such dissipated energy density is expected to be increased by enlarging the bending angle of the crystal, which in this case is 64.28° (Fig. 1a), and/or the required shear stress for the deformation. The high dissipated energy will be effective for the damping of mechanical vibrations.

## Conclusions

We confirmed a new strategy to increase *E*_d_ in organic single crystal, which typically bears smaller stress than those of metallic solids. Large bending angle in twinning ferroelastic deformation enables a wider crystal deformation range for the realization of large energy od dissipation. The relationship between effective bending angle and *E*_d_ can be clearly measured in single crystals without breakage by microscopic and macroscopic experiments. The maximum strain and the *E*_d_ value in 1 is, to the best of our knowledge, the largest reported molecular crystals, facilitated by large deformation but with a smaller shear force. Such controllability paves the way for new design principles to increase the value of *E*_d_ in organic single crystals. The combination of small *σ*_c_ and large *E*_d_ makes organic ferroelastic materials promising for applications in mechanical damping, with high susceptibility to absorb weak shocks effectively by their small volume.

## Conflicts of interest

There are no conflicts to declare.

## Supplementary Material

RA-008-C8RA02499E-s001

RA-008-C8RA02499E-s002

RA-008-C8RA02499E-s003
