# Supplementary material for: Enhancement of dissipated energy by large bending of an organic single crystal undergoing twinning deformation
Source: RSC Adv. 2018 Jun 14;8(39):21933–6. doi: 10.1039/c8ra02499e (PMC9081229; doi:10.1039/c8ra02499e)
Supplement: RA-008-C8RA02499E-s001 [file RA-008-C8RA02499E-s001.pdf]

Electronic Supplementary Material (ESI) for RSC Advances.
This journal is © The Royal Society of Chemistry 2018

Supporting Information

Enhancement of Dissipated Energy by Large Bending of an Organic Single Crystal undergoing Twinning Deformation

Sajjad Husain Mir[a], Yuichi Takasaki[a,b], Emile R. Engel[a], Satoshi Takamizawa[a]*

[a]Department of Materials System Science, Graduate School of Nanobioscience, Yokohama City University, 22-2 Seto, Kanazawa-ku, Yokohama, Kanagawa 236-0027, Japan

[b]Kanagawa Institute of Industrial Science and Technology, Shimoimaizumi, 705-1 Ebina, Kanagawa, 243-0435, Japan

*E-mail: staka@yokohama-cu.ac.jp

Table of contents

(a) Experimental information S2
(b) Crystallographic data S3
(c) Crystal face indexing S4
(d) Detail information for observation of stress-strain test S4
(e) Enlarged color figures described in the main text S5-S6

Other supplementary material

Video file for bending action of **1** observed under a polarizing microscope:

(a) Stress-Strain test at 298 K: Takamizawa_movS1.qt

Materials

2-methyl-5-nitrobenzoic acid was purchased from Tokyo Chemical Industry, Japan. Solvents were purchased from Wako and used as received.

Recrystallization

Crystals of **1** were grown in acetone solution by slow evaporation. A mixture of small and long needle-shaped crystals was obtained.

(a) Experimental information

i) Stress-strain test

Stress tests were carried out on a universal testing machine (Tensilon RTG-1210, A&D Co. Ltd.).

ii) Single-crystal X-ray diffraction experiment

Single-crystal X-ray analysis of **1** was performed at 298 K on a Bruker SMART APEX CCD area detector (graphite-monochromated Mo-Kα radiation (λ = 0.71073 Å)) with a nitrogen flow temperature controller. Empirical absorption corrections were applied using the SADABS program. The structure was solved by direct methods (SHELXS-97) and refined by full-matrix least squares calculations on F^2 (SHELXL-97) using the SHELX-TL program package. Non-hydrogen atoms were refined anisotropically; hydrogen atoms were refined in a riding model. The crystal face indexing was carried out using SMART in a SHELXTL Ver.6.12 program package with a twin resolution program. Crystallographic data of the structure is summarized in Tab. S1.

(b) Crystallographic data

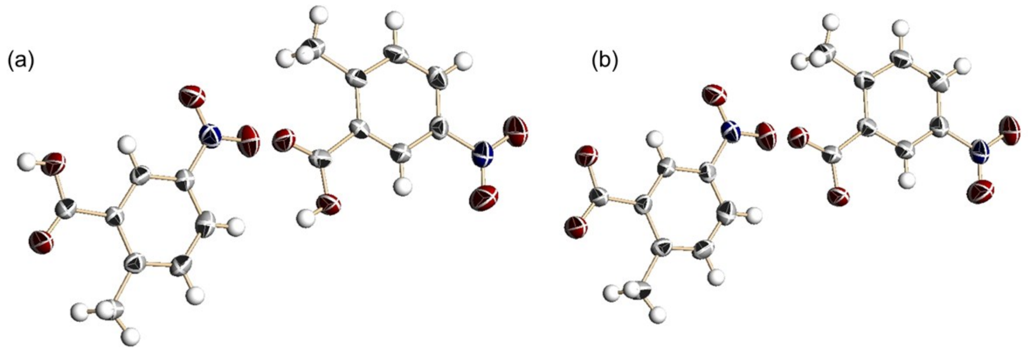

Figure S1. Molecular structures of **1** in (a) mother domain and (b) twinned domain as ORTEP representations drawn at 50% probability level for the ellipsoid obtained from single crystal X-ray diffraction measurement at 298 K.

Table S1. Crystallographic data of **1** in bent shape.

Domain	mother (α_0)	daughter (α_1)
T /K	298	298
Empirical formula	$C_8H_7NO_4$	$C_8H_7NO_4$
M	181.15	181.15
Crystal system	Triclinic	Triclinic
Space group	*P*-1	*P*-1
a /Å	7.611 (15)	7.636 (13)
b /Å	10.47 (2)	10.426 (19)
c /Å	10.55 (2)	10.520 (18)
α /°	89.45 (3)	89.28 (3)
β /°	81.45 (3)	81.80 (3)
γ /°	76.75 (3)	76.32 (3)
V /Å^3	810 (3)	805 (2)
Z	4	4
D_{calc}/Mg m^{-3}	1.485	1.495
μ(Mo Kα) mm^{-1}	0.121	0.122
Reflections collected	2759	2769
Independent reflections (R_{int})	1674	1554
Goodness of fit	1.071	1.161
R_1($I > 2\sigma$ (all data))	0.0601	0.1016
$_wR_2$($I > 2\sigma$ (all data))	0.1786	0.3546
Leastdiff.peak (hole) /eÅ^3	0.233(-0.243)	0.466(-0.431)

(c) Crystal phase indexing

The crystal face indexing showed deformation twinning of **1**. Shear stress formed daughter domain α_1 from α_0 in rotational twinning. The twinning interfaces are $(-21-1)_{\alpha0}//(-21-1)_{\alpha1}$ (or $(-21-1_{\alpha0}//(-21-1)_{\alpha1})$.

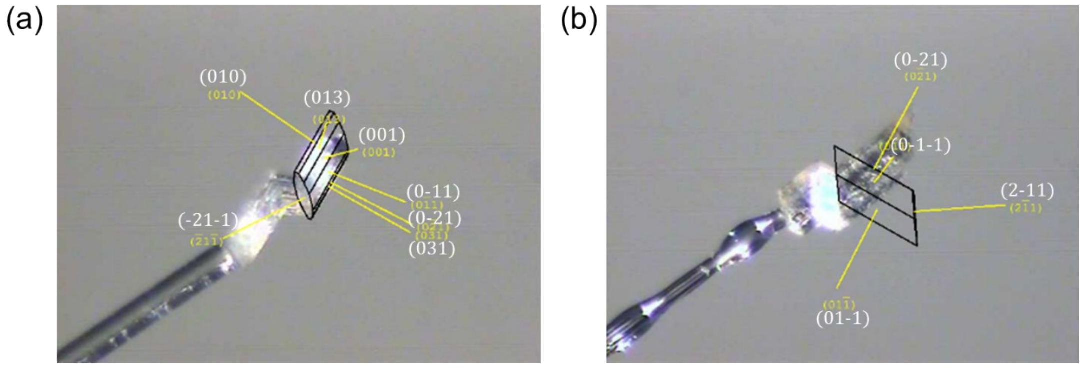

Figure S2. a) Crystal face indices of α_0 domain (a) and α_1 domain (b).

(d) Detail information for observation of stress-strain test

Table S2: Conditions of cyclic shear test on crystal **1**, shown in Fig. 3b

Temperature / °C	Loading surface	Crystal dimension		Displacement Velocity / μm min^{-1}
		width / μm	Thickness / μm	
25	-1-10	32.48	286.96	30

(e) Enlarged color figures described in the main text

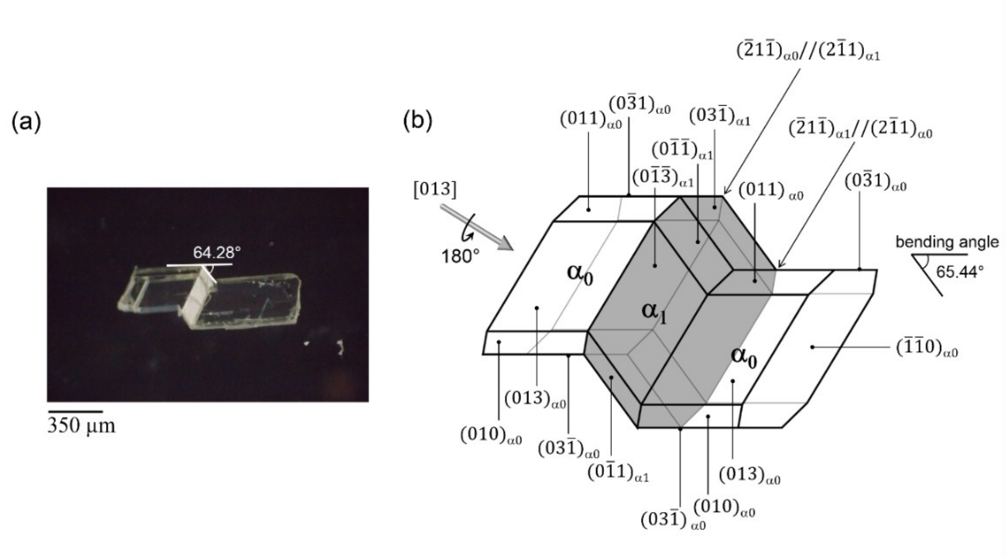

Figure S3. a) Optical image of the twinned crystal by compression on crystal surface (110)α_0, [013] and b) crystal face indices of the mechanically twinned crystal.

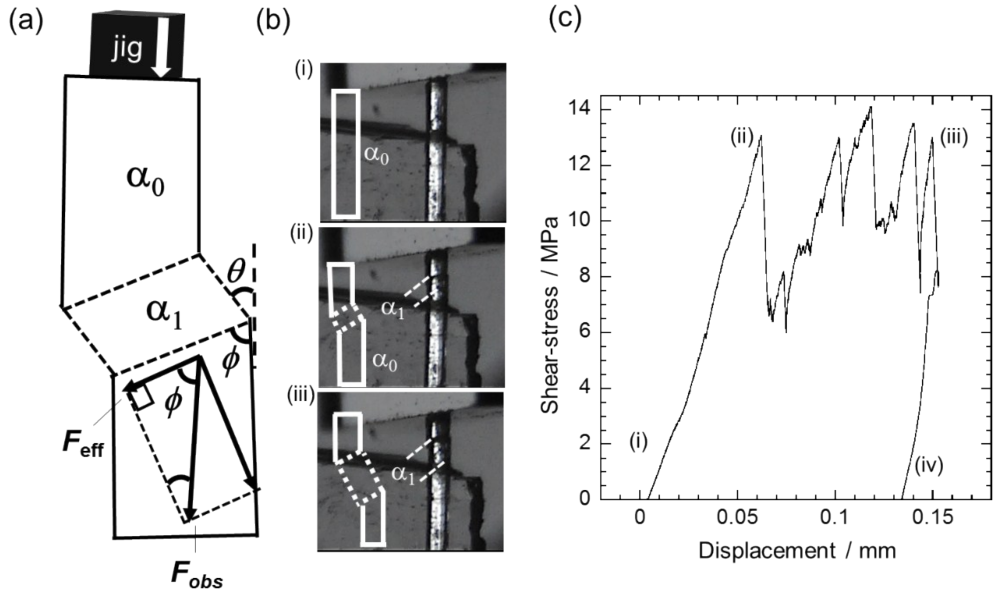

Figure S4. Measurement of stress-strain curve, a) Cartoon illustration of crystal deformation pattern and force components, b) snapshots of the twinning deformation of shear-stress (i-iii) (Movie S1) with inset sketches of the deformation pattern, and c) stress-strain curve at 298 K

Effective stress: σ_{eff}

$F_{eff} = F_{obs} \cos\phi \qquad (\phi = 57.28°)$

$\sigma_{eff} = F_{eff} /$ cross-sectional area

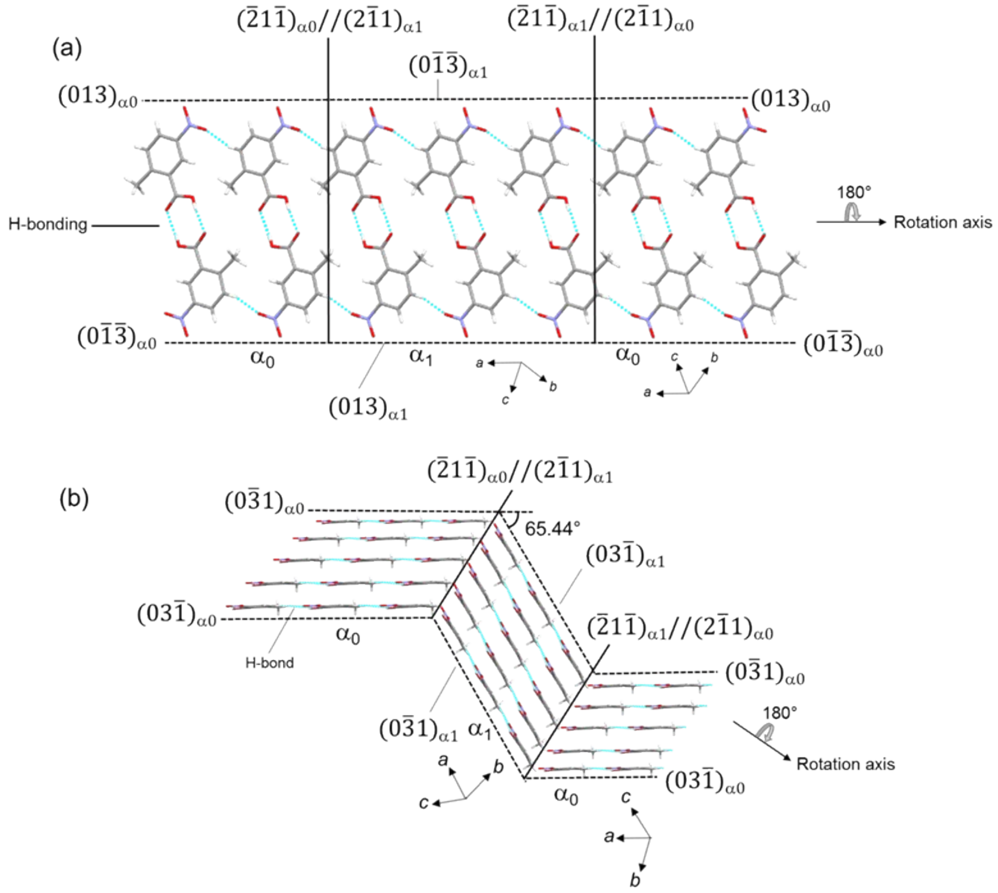

Figure S5. Partial packing diagrams of overlapping mother (α_0) and daughter domain (α_1) of **1** viewed (a) along $[013]_{\alpha0}$ and (b) along $[0\bar{3}1]_{\alpha0}$. Molecules form 2D molecular chains by hydrogen bonding (indicated as dotted lines).
